# Topographic aspect affects the vegetation restoration and artificial soil quality of rock-cut slopes restored by external-soil spray seeding

**DOI:** 10.1038/s41598-018-30651-y

**Published:** 2018-08-14

**Authors:** Ruirui Li, Wenjuan Zhang, Siqian Yang, Mengke Zhu, Shasha Kan, Jiao Chen, Xiaoyan Ai, Yingwei Ai

**Affiliations:** 1 Key Laboratory of Bio-resources and Eco-environment (Ministry of Education), College of Life Sciences, Sichuan University, Chengdu, 610064 China; 2 Key Laboratory of Freshwater Fish Reproduction and Development (Ministry of Education), College of Life Sciences, Southwest University, Chongqing, 400700 China; 3 College of Life Sciences, Sichuan Normal University, Chengdu, 610066 China

## Abstract

External-soil spray seeding (ESSS), a technique of spraying artificial soil materials onto bare slopes for vegetation cover construction, has been widely used to restore rock-cut slopes. However, studies on the effect of the practical application of this technique on different topographic aspects have been rarely performed. In this study, two topographic aspects, namely, north-facing versus south-facing, were investigated under two railway lines, and two local natural slopes (north-facing versus south-facing) were selected as references. Vegetation and soil conditions, which are paramount aspects of ecological restoration assessment, were characterized in terms of the richness and diversity indices, vegetation canopy cover, basic soil physico-chemical properties, and structural characteristics of these slopes. Results showed that (1) the topographic aspect significantly affected the vegetation restoration and artificial soil quality of rock-cut slopes restored by ESSS; (2) the ecological restoration effect of north-facing slopes were better than that of south-facing slopes; and (3) the vegetation and soil conditions of natural slopes were better than those of rock-cut slopes. Therefore, additional scientific management measures should be implemented to promote the ecological restoration of rock-cut slopes, especially for south-facing slopes.

## Introduction

With the rapid growth of the economy in China, major infrastructure projects, such as roads and railways, have been developing faster than ever before. However, these constructions have created abundant bare rock slopes that have had a profound impact on local natural ecosystems^1^; for example, the removal of natural soil and underlying materials^2^, the alteration of landscape patterns^3^, the destruction and degradation of vegetation and biodiversity^4,5^, and the increase in the occurrence of soil erosion and landslides^6^. To restore rock-cut slopes, control surface erosion, and improve landscape aesthetics, researchers have conducted several attempts involving hydraulic covering, geotextile covering, external-soil spray seeding (ESSS), and vegetation concrete foundation spraying^2,7–9^. In southwestern China, ESSS is widely used to restore rock-cut slopes because it is convenient and economical.

ESSS is a technique of spraying artificial soil materials (backfill soil, plant seeds and composite material) onto bare slopes for constructing vegetation cover. Backfill soil is the core of this technique, which provides plants with root anchorage, water, and nutrient sources. Vegetation cover can improve slope soil shear strength, control slope soil erosion, improve soil quality, and facilitate ecosystem restoration^10–12^. Therefore, previous studies on ESSS mainly focused on (1) selecting proper plant seeds for constructing suitable vegetation cover^1,10,13^; (2) improving the viscidity, particle composition, fractal characteristics, and aggregate stability of artificial soils^14–17^; and (3) exploring the impact of artificial soil composition on rock-cut slope nets^18^. However, very little attention has been paid on exploring the practical application effect of this technique on different topographic aspects.

Previous studies confirmed that microclimate, light intensity, soil and air temperature, humidity, soil moisture, and evaporation differ between the two contrasting topographic aspects^19–21^. The topographic aspect, as an important factor, affects the structure, distribution and diversity of vegetation^22^ and the dynamics of carbon, nitrogen, and other nutrients^23,24^. These findings indicated that the topographic aspect directly or indirectly influences abiotic and biotic factors, thereby contributing to the spatial variability of vegetation cover and soil conditions. However, most studies investigating the effects of topographic aspect on soil and plants have been conducted in arid/semiarid zones^23,25^, and catchments^26,27^, and few studies have investigated the reconstituted soils and vegetation in ecological restoration areas^28,29^, especially on railway rock-cut slopes restored by ESSS. ESSS technique, an identical artificial soil mixture is sprayed onto the surface of rock-cut slopes; as such, neglecting the topographic aspect may have a great impact on vegetation growth and artificial soil during ecological restoration. Therefore, the vegetation restoration and artificial soil quality of rock-cut slopes under different topographic aspects must be evaluated and monitored to achieve the successful ecological restoration of rock-cut slopes.

Plant richness and diversity have been used as indicators in the vegetation restoration assessment of ecologically reconstituted areas^16^. The evaluation of vegetation canopy cover is also important because only high vegetation coverage can effectively play the role of protecting slope surfaces^30^. Chen *et al*. (2016) proposed to characterize artificial soil quality by using basic soil physico-chemical and biological properties such as soil organic carbon content, cation exchange capacity, bulk density, and enzyme activities^5^. Huang *et al*. (2017) recommended the assessment of artificial soil quality by considering soil texture, and soil structure^2^. Soil texture and soil structure remarkable affect many soil properties, including water retention, aeration, compaction, porosity, hydraulic conductivity, and nutrient availability^31^. Therefore, these typical indicators were selected to evaluate the vegetation restoration and artificial soil quality of rock-cut slopes with different topographic aspects in the present study. Our main objectives were to evaluate the ecological restoration effect of rock-cut slopes on different topographic aspects and present possible measures to optimize ESSS for improving artificial soils and promoting vegetation restoration. This study could provide a theoretical and practical reference for the ecological restoration of rock-cut slopes.

## Materials and Methods

### Study site characterization

The study site is located near the Suining railway station, Sichuan Province (32° 32′ N, 105° 32′ E). Sichuan Province is located in southwestern China. Its terrain is complex and diverse, including plains, plateaus, mountains, and hills. The study site, which is at the center of the Sichuan Basin, is a mainly hilly and low mountain area. The hilly region is mainly modulated by erosional and accumulation processes. The lower part of the soil rock layer is mainly limestone, and the upper part is mostly purple soil and mudstone, which belongs to a typical Jurassic strata. It has a typical subtropical humid monsoon climate with an annual average wind velocity of 0.7 m/s, an annual average temperature of 17.4 °C, and an annual average rainfall of 927.6 mm. The soil at the study site was classified as Eutric Cambisol according to the FAO–UNESCO system.

The southwestern region has a subtropical monsoon climate, and heavy rains often occur during the monsoon season from June to September, resulting in frequent landslides^32^. However, the construction of railways at this study site inevitably produced many unstable rock-cut slopes, and therefore, the potential risks such as landslides and collapses are also large. In order to reduce railway disasters and ensure the safety of railway, external-soil spray seeding (ESSS) was applied to promote the ecological restoration of these unstable rock-cut slopes. The general technical flow of this technology is: (1) mixing backfill soil, plant seeds and other materials in an appropriate proportion, (2) installing rivets and fixing piles, (3) covering with a protective net (barbed wire), (4) spraying the artificial soil mixture onto the rock-cut slope using a sowing machine, and (5) laying the non-woven to the rock-cut slope. The artificial soil used by ESSS is composed of humus (6.25 kg/m^2^), straw (0.1 kg/m^2^), soluble chemical fertilizer (N:P_2_O_5_:K_2_O = 16:6:8; 0.3 kg/m^2^), composite material (bonding and water-retention agents; 0.225 kg/m^2^), seed mixture (0.03 kg/m^2^), backfill soil (rock fragments, obtained from the rock-cut slope surface after crushing and passing through 2 mm sieves).

### Experimental design

**Figure 1:**
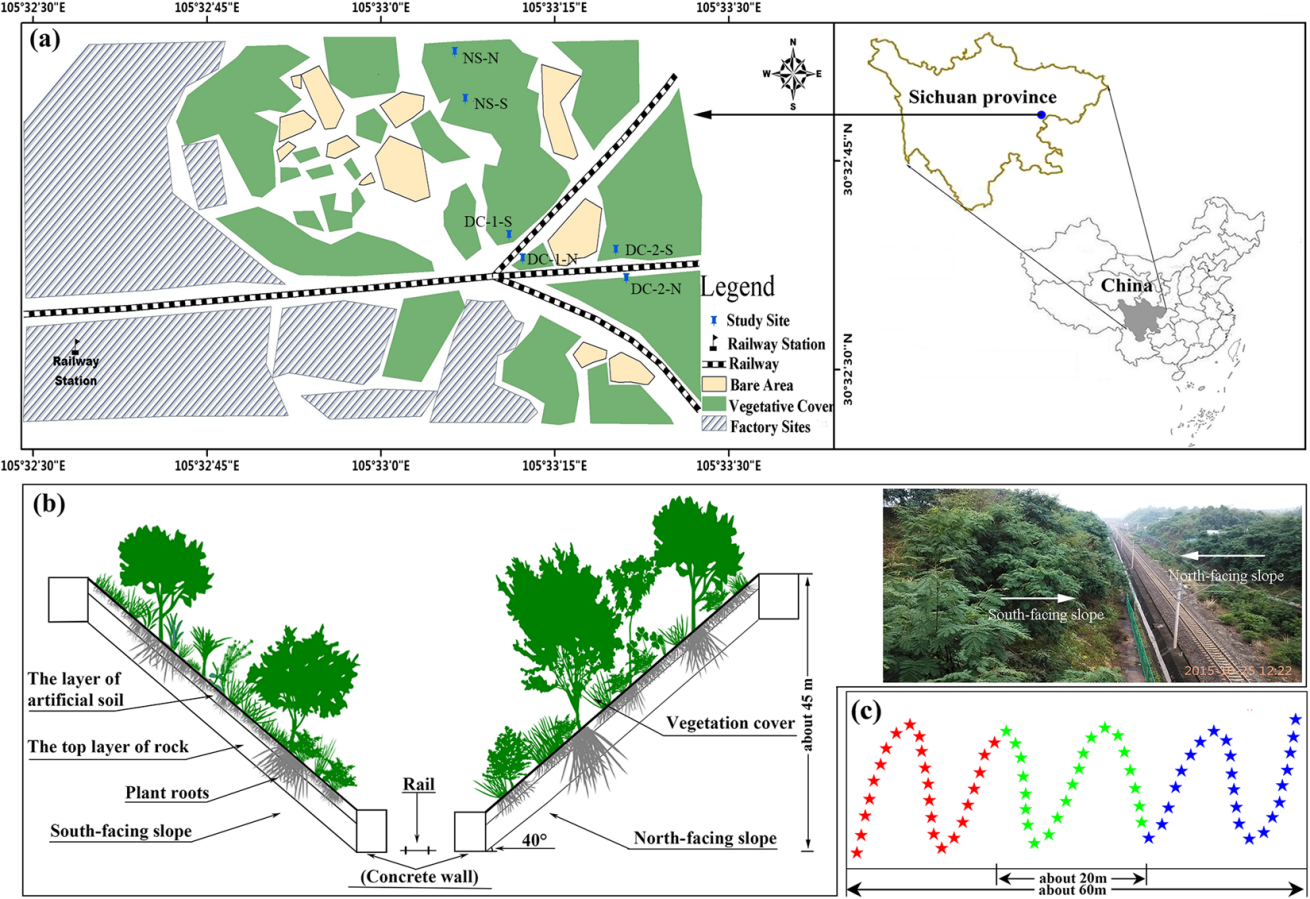
Detailed locations of the study site and sampling strategy. Notes: (**a**) Study sites. DC-1-N: Dazhou-Chengdu line–north-facing slope; DC-1-S: Dazhou-Chengdu line south-facing slope; DC-2-N: Dazhou-Chengdu second line–north-facing slope; DC-2-S: Dazhou-Chengdu second line–south-facing slope; NS-N: natural–north-facing slope; NS-S: natural–south-facing slope. The main part of this map was generated by the co-author (Yingwei Ai) using the ArcGIS software (version 10.0, ESRI Inc.; Redlands, CA, USA; homepage: https://www.esri.com/), and a part of this map was quoted from an article published by Huang *et al*.^2^. (**b**) Sectional drawing of rock-cut slopes. Plants in the drawing do not represent the actual growth of plants in sample areas. The photograph was taken by one of the authors (Jiao Chen). (**c**) Sampling strategy. Samples were collected from red stars and mixed together to obtain a homogenized weight sample of approximately 2 kg (replicate 1), as did green stars (replicate 2) and blue stars (replicate 3).

Four rock-cut slopes restored by ESSS (identical artificial soil) from two railway lines (Dazhou-Chengdu line and Dazhou-Chengdu second line) and two natural slopes were selected in this study, as shown in Fig. 1a: (1) Dazhou-Chengdu line–north-facing slope (DC-1-N) and Dazhou-Chengdu line–south-facing slope (DC-1-S), restored in 1996; (2) Dazhou-Chengdu second line–north-facing slope (DC-2-N) and Dazhou-Chengdu second line–south-facing slope (DC-2-S), restored in 2007; (3) Natural–north-facing slope (NS-N) and natural–south-facing slope (NS-S) without human disturbances. NS-N and NS-S were used as references. The height of the selected slopes was approximately 45 m, and inclined by approximately 40° (Fig. 1b).

### Soil sampling and vegetation survey

A simple experimental design with six treatments and three samples per treatments was collected through an S-shaped sampling strategy (Fig. 1c). Each soil sample (approximately 2 kg) comprised soils collected from twenty-five to thirty points. The soil surface litter was removed, and then a shovel was used to take soil samples. The artificial soil thickness of the rock-cut slopes restored by ESSS was approximately 10 cm, and therefore, sampling depth was 0–8 cm for all the slopes. All soil samples were collected in October 2015.

Before soil sampling, the plant richness, diversity and vegetation canopy cover were investigated to evaluate the effects of topographic aspect on the vegetation restoration of rock-cut slopes. Five large test fields (5 m × 5 m) and ten small test fields (1 m × 1 m) were randomly placed on each slope for investigating the Margalef index (*D*) and Shannon-Wiener index (*H*′) of herbs, trees and shrubs. The equations were as follows:

1 $$D=\frac{(S-1)}{\mathrm{ln}\,N}$$

2 $$H\text{'}=-\,\sum _{i=1}^{S}{P}_{i}\,\mathrm{ln}\,{P}_{i}$$

where *S* was the total number of species in a community, *N* was the total number of individuals in a community, and *P*_*i*_ was the percentage of the number of one plant to the total number of all plants.

Vegetation canopy cover was estimated by analysing photographs from the test fields. The main steps were: (1) we put the camera in the middle of these test fields and took photos; (2) the photos were processed with Adobe Photoshop CS6 Extend 13.0, and 15 × 10 square meshes (each small square side length for 1 unit) were created; (3) the 15 × 10 square meshes were embedded into the photos to form a combination chart; and (4) the number of square meshes representing plant and sky area were counted (plant area, which occupies more than half of a small square, was considered to have occupied all of the small squares). Vegetation canopy cover was estimated as follows:

3 $${\rm{Vegetation}}\,{\rm{canopy}}\,{\rm{cover}}\,( \% )=\frac{100\ast {\rm{Number}}\,{\rm{of}}\,{\rm{plant}}\,{\rm{square}}\,{\rm{meshes}}}{{\rm{Number}}\,{\rm{of}}\,({\rm{plant}}+{\rm{sky}})\,{\rm{square}}\,{\rm{meshes}}}$$

### Determination of soil physico-chemical properties and enzyme activity

The oven drying method (105 °C, 24 h) was used to determine soil water content (SWC) immediately after field sampling^33^. Bulk density (BD) was estimated using the oven-dried soil sample mass and volume based on the core method (stainless steel cylinders with a volume of 100 cm^3^)^34^. Then the other soil samples was placed in the laboratory for natural air-drying after picking out any gravel, animal and plant debris. The air-dried soil samples were ground and then passed through a 20 and 100 mesh nylon sieve. The processed samples were preserved for the determination of soil organic carbon (SOC), soil pH, as well as other biochemical parameters.

Soil pH was determined by the Glass Calomel Electrode^35^. SOC was measured by the potassium dichromate oxidation heating method^36^. Available phosphorus (AP) was assayed by the Olsen method^37^. Available potassium (AK) was assayed by the flame photometry method^38^. Available nitrogen (AN) was assayed using a micro-diffusion technique after alkaline hydrolysis^39^. The activities of Protease, Sucrase, Catalase and Urease were measured following Zhang *et al*.^40^ and Hu *et al*.^41^.

All experiments were completed in the laboratory of the College of Life Sciences of Sichuan University.

### Determination of soil structure parameters

The particle size distribution (PSD) (<0.002, 0.002–0.005, 0.005–0.02, 0.02–0.05, 0.05–0.25, and 0.25–2 mm) was measured using the pipette method after the removal of organic matter using hydrogen peroxide and heat treatments, with sodium hydroxide as the dispersing agent^16^. Soil texture class [clay content (<0.002 mm), silt content (0.002–0.02 mm), and sand content (0.02–2 mm)] was identified by using the International Society of Soil Science (ISSS) soil texture classification system^5,42,43^. Fractal dimension (*D*_m_) was used to describe the distribution of soil particles^44^, the equation were as follow:

4 $$\mathrm{log}[W(r < \overline{{r}_{i}})/{W}_{T}]=(3-{D}_{{\rm{m}}})\,\mathrm{log}(\overline{{r}_{{\rm{i}}}}/\overline{{r}_{{\rm{\max }}}})$$

where $\overline{{r}_{i}}$ is the mean particle diameter (mm) of the *i*th size class, that is, the arithmetic mean of the upper and lower sieve sizes; $\overline{{r}_{{\rm{\max }}}}$ is the mean diameter (mm) of the largest size class*; W* is the cumulative weight (g) of the particles with diameters of less than $\overline{{r}_{i}}$; *W*_*T*_ is the total weight (g) of the particles. Simple linear regression analysis was used to establish the relationships between $\mathrm{log}[W(r < \overline{{r}_{i}})/{W}_{T}]$ and $\mathrm{log}(\overline{{r}_{{\rm{i}}}}/\overline{{r}_{{\rm{\max }}}})$, yielding a linear equation of the form “*y = ax + b*”. The slope of the line, *a*, is equal to “3 − *D*_m_”. Thus, *D*_m_ can be calculated using the equation “*D*_m_* = *3 − *a*”.

The structural failure rate of soil aggregates (P) and area difference (∆S) were selected to characterize the soil aggregate stability and erosion durability, and these two indicators were calculated according to following equation:

5 $${\rm{P}}=\frac{a-b}{a}$$

where P is the structural failure rate of soil aggregates; *a* is the percentage of aggregates >0.25 mm, measured by Yoder’s method, dry sieving; and *b* is the percentage of water-stable aggregates >0.25 mm, measured by Yoder’s method, wet sieving.

6 $$\begin{array}{c}{\rm{\Delta }}S={{\rm{S}}}_{{\rm{wet}}}-{{\rm{S}}}_{dry}={\int }_{\overline{{r}_{{\rm{\min }}-{\rm{wet}}}}}^{\overline{{r}_{{\rm{\max }}-{\rm{wet}}}}}f({r}_{{\rm{wet}}})d{r}_{{\rm{wet}}}-{\int }_{\overline{{r}_{{\rm{\min }}-{\rm{dry}}}}}^{\overline{{r}_{{\rm{\max }}-{\rm{dry}}}}}f({r}_{{\rm{dry}}})d{r}_{{\rm{dry}}}\\ \,\,=\,F({r}_{{\rm{wet}}})|\frac{\overline{{r}_{{\rm{\max }}-{\rm{wet}}}}}{\overline{{r}_{{\rm{\min }}-{\rm{wet}}}}}-F({r}_{{\rm{dry}}})|\frac{\overline{{r}_{{\rm{\max }}-{\rm{dry}}}}}{\overline{{r}_{{\rm{\min }}-{\rm{dry}}}}}\end{array}$$

7 $$f({r}_{{\rm{wet}}/{\rm{dry}}})=a{{r}_{\mathrm{wet}/\mathrm{dry}}}^{2}+b{r}_{\mathrm{wet}/\mathrm{dry}}+c$$

where ∆S is the area difference of cumulative distribution of soil aggregate weights after wet sieving and dry sieving. S_wet_ and S_dry_ are the areas of wet sieving curves and dry sieving curves, respectively; $\overline{{r}_{max-wet}}$ and $\overline{{r}_{max-dry}}$ are the mean particle diameters (mm) of the *r*_max_ size class under wet sieving and dry sieving, respectively; $\overline{{r}_{{\rm{\min }}-{\rm{wet}}}}$ and $\overline{{r}_{{\rm{\min }}-{\rm{dry}}}}$ are the mean particle diameters (mm) of the *r*_min_ size class under wet sieving and dry sieving, respectively; *r*_wet/dry_ is the mean particle diameter (mm) of different size classes under wet sieving or dry sieving. *f* (*r*_wet/dry_) is a quadratic function; and the *a, b* and *c* are constants (*a* ≠ 0).

### Statistical analysis

Data were collected from the study sites involving four railway rock-cut slopes (DC-1-N, DC-1-S, DC-2-N and DC-2-S) and two natural slopes (NS-N and NS-S). Linear regression analysis was used for calculating the fractal dimensions of PSD (*D*_m_) and exploring the relationship of *D*_m_ and soil clay, silt, sand content. The differences in the soil variables and vegetation parameters under different slopes were analysed using analysis of variance (ANOVA) with least significant difference (LSD). Pearson correlation matrix analysis (CMA) was used to analyse the relationships between selected soil properties. Principal component analysis (PCA) was used to determine the crucial factors among selected soil variables. ANOVA, CMA and PCA were performed using SPSS 19.0. A level of P < 0.05 was considered to be significant.

## Results

### Vegetation and fundamental soil parameters

**Figure 2:**
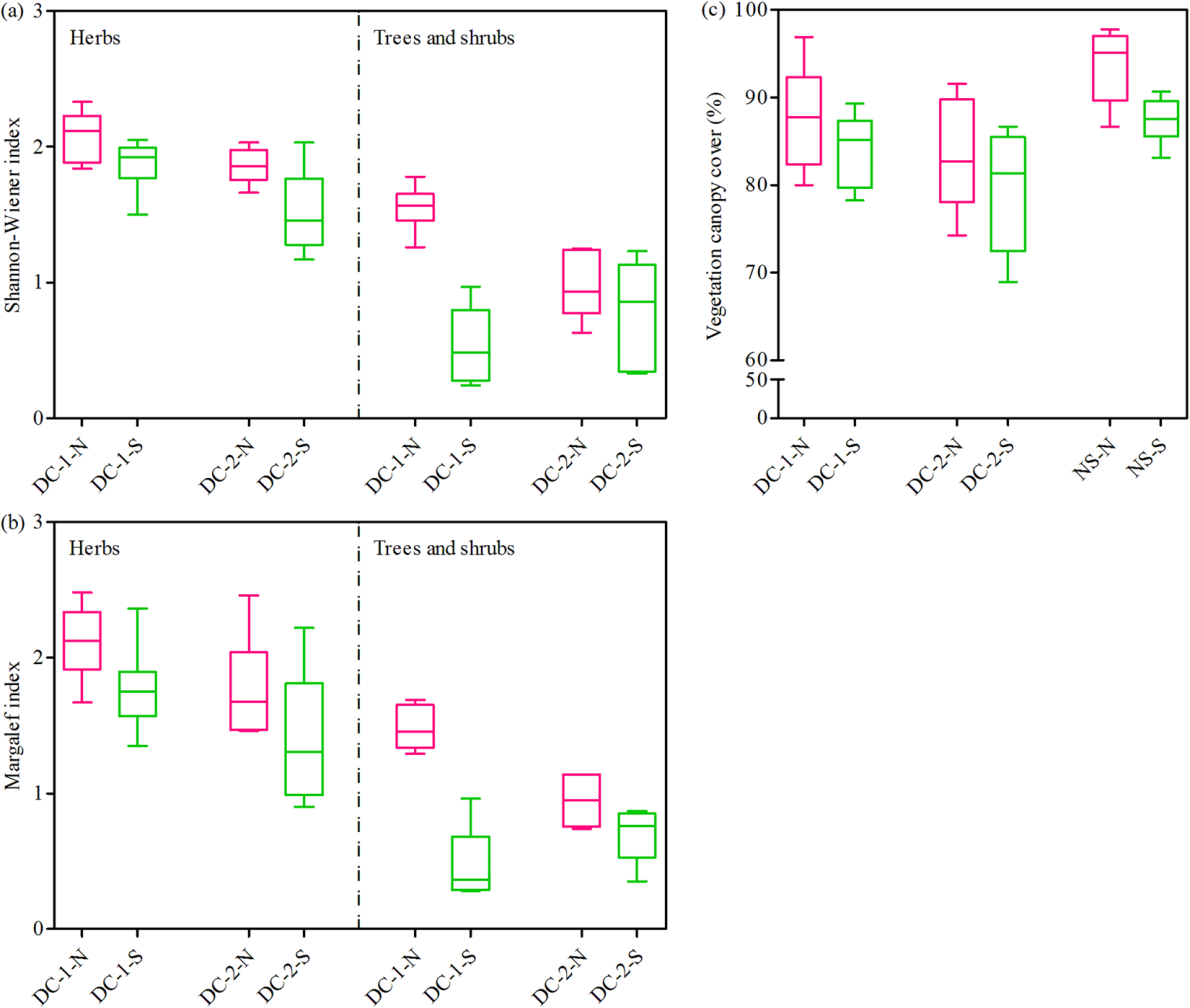
The canopy coverage, Margalef index, and Shannon-Winner index of slopes. Notes: DC-1-N: Dazhou-Chengdu line–north-facing slope; DC-1-S: Dazhou-Chengdu line–south-facing slope; DC-2-N: Dazhou-Chengdu second line–north-facing slope; DC-2-S: Dazhou-Chengdu second line–south-facing slope; NS-N: natural–north-facing slope; NS-S: natural–south-facing slope. The Margalef index and Shannon-Winner index of natural slopes were not investigated and counted.

Figure 2 shows that the Shannon-Wiener index (herbs; trees and shrubs) between the north- and south-facing slopes considerably differed; that is, the index of the former was higher than that of the latter (DC-1-N > DC-1-S; DC-2-N > DC-2-S). The Margalef index (herbs; trees and shrubs) exhibited the same spatial trend. The vegetation canopy cover of the north-facing slopes was also significantly higher than that of the south-facing slopes. However, the vegetation canopy cover of the cut slopes was obviously lower than that of the natural slopes: DC-2-N < DC-1-N < NS-N; DC-2-S < DC-1-S < NS-S. These results suggested that (1) the topographic aspect significantly affected the vegetation restoration performance; (2) the vegetation condition on the north-facing slopes was better than that on the south-facing slopes; and (3) the vegetation conditions of the cut slopes were poorer than those of the natural slopes after many years of ecological restoration.

**Figure 3:**
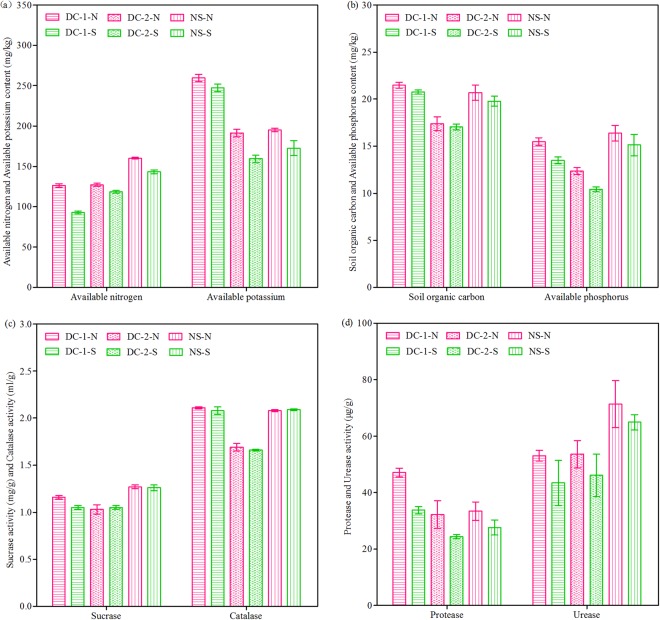
Selected basic soil chemical properties and enzyme activities of slope soils. Notes: DC-1-N: Dazhou-Chengdu line–north-facing slope; DC-1-S: Dazhou-Chengdu line–south-facing slope; DC-2-N: Dazhou-Chengdu second line–north-facing slope; DC-2-S: Dazhou-Chengdu second line–south-facing slope; NS-N: natural–north-facing slope; NS-S: natural–south-facing slope.

Table 1 shows some basic soil properties, including bulk density (BD), soil water content (SWC), and pH, which are often used as indicators of artificial soil quality^2^. The BD of the north-facing slopes was lower than that of the south-facing slopes. By contrast, the SWC of the north-facing slopes were significantly higher than that of the south-facing slopes. The pH showed slight differences between the two contrasting topographic aspects, with a comparatively lower value in the north-facing slopes. Some soil nutrients and enzyme activities, such as soil organic carbon (SOC), available nitrogen (AN), available phosphorus (AP), available potassium (AK), protease, urease, sucrase, and catalase, are also usually considered as indicators of soil quality^2,16,45,46^. The data of these properties in all rock-cut slopes under the two railway lines are shown in Fig. 3. The contents of SOC, AN, AP, and AK are also significantly varied between the two contrasting aspects. In particular, the nutrient content on the north-facing slopes was higher than that on the south-facing slopes. Consistent with these results, the protease, urease, sucrase, and catalase activities on the north-facing slopes were higher than those on the south-facing slopes, indicating that the ecological environment of the former was better than that of the latter. The SWC, AN, AP, sucrase activity, and urease activity on the natural slopes were significantly higher than those on the cut slopes (DC-2-N < DC-1-N < NS-N; DC-2-S < DC-1-S < NS-S). However, some properties, such as BD and pH, of the natural slopes were obviously lower than those of the cut slopes (DC-2-N > DC-1-N > NS-N; DC-2-S > DC-1-S > NS-S). These results implied that soil conditions remarkably differed between cut slopes and natural slopes.

**Table 1:** Selected basic soil properties and characteristic parameters of particle size distribution and aggregate stability.

Topographic aspect	Variables	Dazhou-Chengdu line	Dazhou-Chengdu second line	Natural slopes
North-facing	BD (g·cm^−3^)	1.282 ± 0.020Ab	1.339 ± 0.010Aa	1.240 ± 0.023Ac
	SWC (%)	19.92 ± 0.11Aa	17.30 ± 0.34Ab	20.65 ± 1.64Aa
	pH	7.88 ± 0.03Ba	7.78 ± 0.01Aa	7.60 ± 0.12Bb
	Clay (%)	12.07 ± 1.58Ab	9.70 ± 0.47Ac	15.97 ± 0.44Aa
	Silt (%)	28.59 ± 2.62Ab	19.16 ± 2.09Bc	32.83 ± 0.96Aa
	Sand (%)	62.79 ± 3.18Ab	71.14 ± 2.07Aa	51.20 ± 1.06Bc
	Texture class	Sandy Loam	Sandy Loam	Clay Loam
	*D*_m_	2.716 ± 0.009Ab	2.694 ± 0.003Ac	2.753 ± 0.005Aa
	*a* (%)	96.96 ± 0.40Aa	95.06 ± 0.83Aa	96.21 ± 0.19Aa
	*b* (%)	76.76 ± 0.61Ab	72.58 ± 0.12Ac	81.92 ± 0.69Aa
	P (%)	20.83 ± 0.65Bb	23.65 ± 0.62Ba	14.85 ± 0.87Bc
South-facing	BD (g·cm^−3^)	1.293 ± 0.024Ab	1.357 ± 0.025Aa	1.257 ± 0.002Ac
	SWC (%)	17.78 ± 0.60Bb	16.05 ± 0.43Bc	19.29 ± 0.50Ba
	pH	7.91 ± 0.04Aa	7.81 ± 0.02Ab	7.84 ± 0.02Ab
	Clay (%)	11.54 ± 0.69Aa	8.31 ± 1.22Bb	12.13 ± 1.59Ba
	Silt (%)	26.99 ± 0.27Bb	24.52 ± 0.65Ac	28.73 ± 2.64Ba
	Sand (%)	61.47 ± 0.72Ab	67.17 ± 1.49Ba	59.14 ± 2.04Ab
	Texture class	Sandy Loam	Sandy Loam	Sandy Loam
	*D*_m_	2.685 ± 0.011Ab	2.686 ± 0.014Ab	2.723 ± 0.012Ba
	*a* (%)	96.05 ± 0.54Aa	90.81 ± 0.44Bb	94.82 ± 0.05Aa
	*b* (%)	70.88 ± 0.95Bb	61.70 ± 0.25Bc	77.24 ± 0.47Ba
	P (%)	26.20 ± 1.29Ab	32.06 ± 0.33Aa	18.54 ± 0.47Ac

Notes: Numbers followed by different first capital letters within the same column have significant differences (P < 0.05) between different topographic aspects (north-facing versus south-facing), the same sample area for same variables (BD, SWC, pH, clay, silt, sand, *D*_m_, *a*, *b* and P). Numbers followed by different second lowercase letters within the same line have significant differences (P < 0.05) between different sample areas, the same topographic aspect for same variables (BD, SWC, pH, clay, silt, sand, *D*_m_, *a*, *b* and P).

Data are expressed as mean values ± standard deviation with n = 3.

BD: bulk density; SWC: soil water content; *D*_m_: fractal dimension; *a*: percentages of soil *a*ggregates >0.25 mm; *b*: percentages of soil water-aggregates >0.25 mm; P: structure failure rate.

### Soil texture, fractal dimension, and aggregate stability

**Figure 4:**
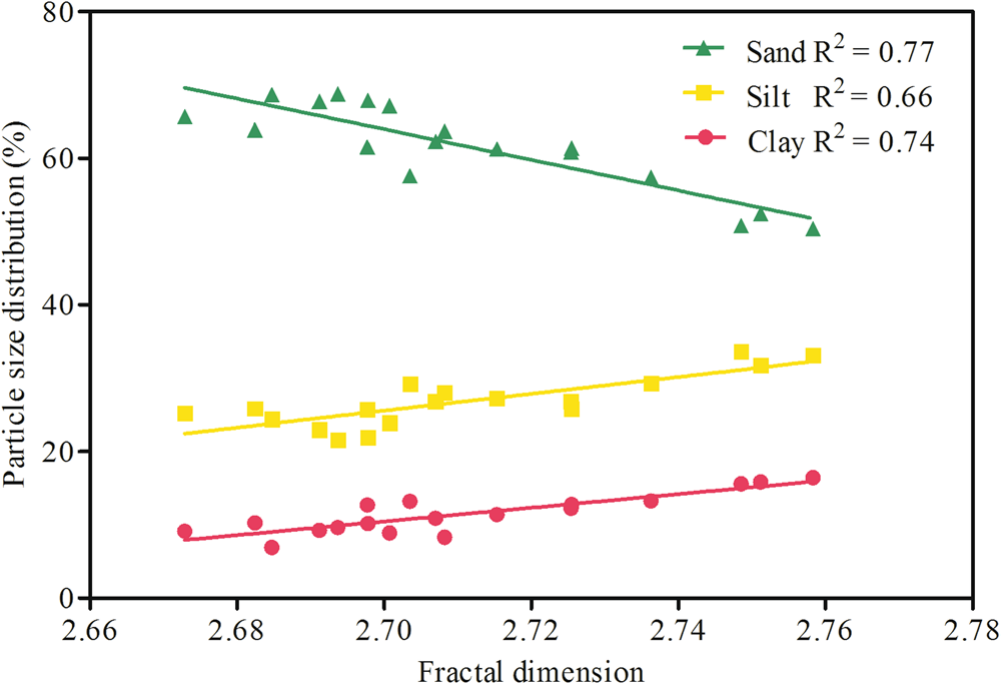
Linear regression analysis for fractal dimension and particle size distribution. , sand, *y* = –208.81*x* + 627.75; , silt, *y* = 115*x* – 284.93; , clay, *y* = 93.759*x* – 242.68.

As shown in Table 1, significant differences were found in soil particle size distribution (PSD) between north- and south-facing slopes. In comparison with the south-facing slopes, the north-facing slopes had more clay content and less sand content. In terms of texture, NS-N was clay loam, whereas the other slopes were sandy loam. Table 1 also shows the fractal dimension (*D*_m_) of the slopes. The values of DC-1-N and DC-2-N were higher than those of DC-1-S and DC-2-S, respectively. However, DC-1-N and DC-2-N had lower *D*_m_ than that of NS-N and NS-S, respectively. Figure 4 shows that *D*_m_ was positively correlated with fine particle content (clay + silt content) and negatively correlated with coarse particle content (sand content). The determination coefficients (R2) of linear regressions were high, ranging from 0.66 to 0.77. These results suggested that the fractal model could be used to evaluate the PSD of artificial soils.

**Figure 5:**
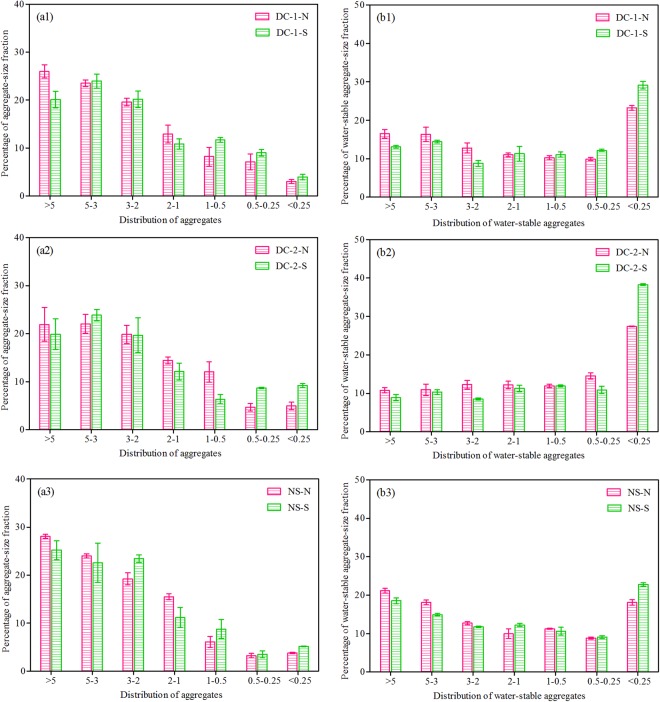
Percentage of soil aggregate fraction and water-stable aggregate fraction. Notes: DC-1-N: Dazhou-Chengdu line–north-facing slope; DC-1-S: Dazhou-Chengdu line–south-facing slope; DC-2-N: Dazhou-Chengdu second line–north-facing slope; DC-2-S: Dazhou-Chengdu second line–south-facing slope; NS-N: natural–north-facing slope; NS-S: natural–south-facing slope.

The detailed aggregate fractions are shown in Fig. 5(a1–a3). More macro-aggregates were found in the north-facing slopes than in the south-facing slopes. The variation in the water-stable aggregate fraction was not consistent with the aggregate fraction [Fig. 5(b1–b3)]. Small water-stable aggregates were dominant, and their percentage on the north-facing slopes was lower than that of the south-facing slopes. This finding indicated that the topographic aspect affected not only the distribution of aggregates but also the distribution of water-stable aggregates. More macro-aggregates ( > 0.25 mm, dry sieving) (DC-2-N < DC-1-N < NS-N; DC-2-S < DC-1-S < NS-S) and lesser small water-stable aggregates (<0.25 mm, wet sieving) (DC-2-N > DC-1-N > NS-N; DC-2-S > DC-1-S > NS-S) were observed in the natural slopes than in the cut slopes. These results suggested that the soil erosion durability of the north-facing slopes was greater than those of the south-facing slopes but was still worse than that of the natural slopes.

**Figure 6:**
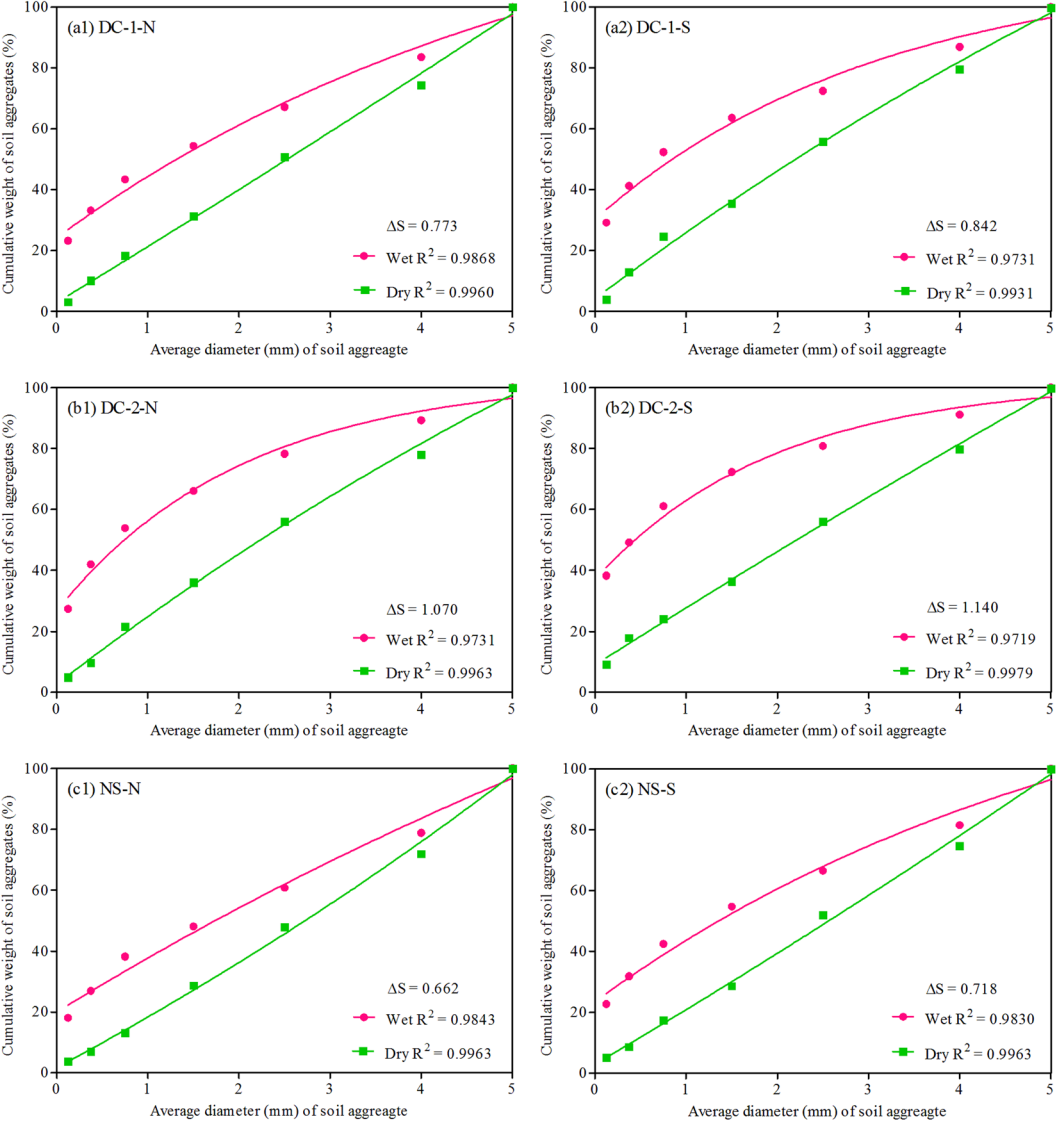
Cumulative distribution of soil aggregate weights after dry sieving and wet sieving. Notes: DC-1-N: Dazhou-Chengdu line–north-facing slope; DC-1-S: Dazhou-Chengdu line–south-facing slope; DC-2-N: Dazhou-Chengdu second line–north-facing slope; DC-2-S: Dazhou-Chengdu second line–south-facing slope; NS-N: natural–north-facing slope; NS-S: natural–south-facing slope. ∆S: area difference between the dry sieving and wet sieving curves.

Soil aggregate stability increased as the structural failure rate and area difference (∆S) between dry- and wet-sieved curves decreased. The structural failure rates and ∆S of the slopes are shown in Table 1 and Fig. 6. The structural failure rates of DC-1-N and DC-2-N were obviously lower than those of DC-1-S and DC-2-S, respectively. A significantly lower ∆S was found in DC-1-N and DC-2-N than in DC-1-S and DC-2-S, respectively. Additionally, the structural failure rate and ∆S of the cut slopes were significantly higher than those of the natural slopes. These results indicated that the aggregate stability of the north-facing slopes was higher than that of the south-facing slopes but was worse than that of the natural slopes. These observations were consistent with the results obtained from macro-aggregates and water-stable aggregates.

### Correlation analysis and principal component analysis

**Figure 7:**
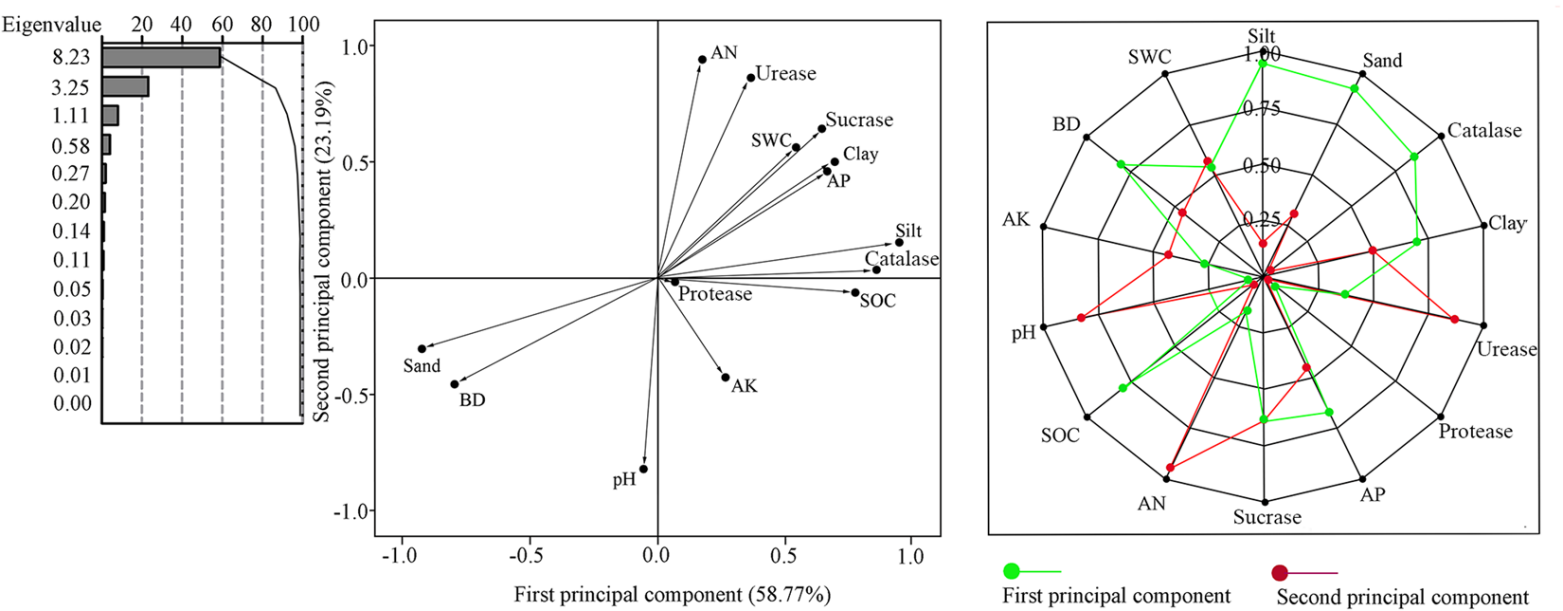
Principal component analysis of soil properties, and first two component’s capacity score coefficients. AN: available nitrogen; AP: available phosphorus; AK: available potassium; SOC: soil organic carbon; BD: bulk density; SWC: soil water content.

The soil nutrients were significantly correlated with fundamental soil parameters and enzyme activities (Table 2). Soil PSD and aggregate stability were also significantly correlated with some soil nutrients (Table 3). Principal component analysis after VARIMAX rotation generated multiple principal components, but only three principal components had eigenvalues of >1.00 (Fig. 7). Among them, the first principal component accounted for 58.77% of the total variation. The primary mineral particles (i.e., silt, clay, and sand) and the SOC had a higher factor loading than the other factors within this principal component, indicating that these variables were highly sensitive in response to soil improvement and implicated in soil restoration.

**Table 2:** Pearson correlation coefficients for soil nutrient, enzyme activities and basic soil properties.

	Protease	Urease	Sucrase	Catalase	BD	pH	SWC
AN	−0.047	0.841^**^	0.792^**^	0.647^**^	−0.551^*^	−0.735^**^	0.608^**^
AP	0.529^**^	0.636^**^	0.795^**^	0.848^**^	−0.861^**^	−0.206	0.891^**^
AK	0.818^**^	−0.293	−0.102	0.583^*^	−0.220	0.415	0.327
SOC	0.619^**^	0.208	−0.541^*^	0.936^**^	−0.713^**^	0.134	0.685^**^

Notes: AN: available nitrogen; AK: available potassium; AP: available phosphorus; SOC: soil organic carbon; BD: bulk density; SWC: soil water content. ^**^Correlation is significant at P < 0.01; ^*^Correlation is significant at P < 0.05.

**Table 3:** Pearson correlation coefficients among characteristic parameters and selected soil properties.

	Clay	Silt	Sand	AP	AK	AN	SOC	P	*D*_m_
Clay	1								
Silt	0.683^**^	1							
Sand	−0.864^**^	−0.958^**^	1						
AP	0.838^**^	0.645^**^	−0.774^**^	1					
AK	0.211	0.170	−0.200	0.379	1				
AN	0.523^*^	0.330	−0.434	0.547^*^	−0.410	1			
SOC	0.647^**^	0.706^**^	−0.741^**^	0.805^**^	0.720^**^	0.086	1		
P	−0.784^**^	−0.540^*^	0.680^**^	−0.898^**^	−0.146	−0.754^**^	−0.684^**^	1	
*D*_m_	0.810^**^	0.646^**^	0.764^**^	0.770^**^	0.045	0.776^**^	0.561^*^	−0.854^**^	1

Notes: AN: available nitrogen; AP: available phosphorus; AK: available potassium; SOC: soil organic carbon; P: structure failure rate; *D*_m_: fractal dimension. ^**^Correlation is significant at P < 0.01; ^*^Correlation is significant at P < 0.05.

## Discussion

### Topographic aspect affects vegetation and fundamental soil parameters

Vegetation restoration is the core objective of the ecological rehabilitation of rock-cut slopes. Vegetation is highly effective at controlling soil erosion processes and reducing the risk of overland flow generation^47^. In our study, the Shannon-Wiener index, the Margalef index, and the vegetation canopy cover of the north-facing slopes were higher than those of the south-facing slopes. This result implies that the vegetation conditions of the north-facing slopes were better than those of the south-facing slopes after many years of ecological restoration. Furthermore, the former were also more able to reduce soil erosion than the latter after many years of ecological restoration. Soil water is a major driving force of plant community succession and has a significant effect on plant species richness and diversity^48,49^. Consistent with previous findings^19^, our results showed that the SWC content of the north-facing slopes was significantly different from that of the south-facing slopes, and this observation might be the main factor that caused the differences in the Margalef index, Shannon-Wiener index, and vegetation canopy cover between the two contrasting aspects. Pearson’s correlation analysis demonstrated that BD exhibited a significantly negative correlation with soil nutrients, pH showed a significantly negative correlation with AN, and SWC presented a significantly positive correlation with soil nutrients, such as AN, AP, and SOC (Table 2). A higher SWC and lower BD and pH were detected in the north-facing slopes than in the south-facing slopes, so the former likely had a high soil nutrient content and were conducive to plant growth. This phenomenon might be another reason for the higher Shannon-Wiener index, Margalef index, and vegetation cover in the north-facing slopes than in the south-facing slopes.

Some soil nutrients and activities of enzymes, such as SOC, AN, AP, AK, protease, urease, sucrase, and catalase, are usually considered as indicators of soil quality^2,16,45,46^. Thus, we chose these properties to characterize the artificial soil quality of rock-cut slopes and found that the values of these properties in the north-facing slopes were higher than those in the south-facing slopes, indicating high soil quality and good ecological environment. This may be attributed to north-facing slopes having higher SWC, and lower BD and pH, as mentioned above. This may also be due to north-facing slopes having higher vegetation canopy cover, richness, and diversity indices after many years of ecological restoration (Fig. 2), thus producing more litter. The increase in plant litter directly leads to an increase in the input of soil carbon, nitrogen, and other nutrient elements^1,50,51^. In our study, the protease, urease, sucrase, and catalase activities in the north-facing slopes were significantly different from those in the south-facing slopes. The topographic aspect determines solar radiation, which affects enzyme activity. We inferred that this relation was affected by the soil microenvironment as supported by Ren *et al*.^52^ and Baldrian *et al*.^53^. Additionally, high correlation coefficients were found between enzyme activities and nutrient content (Table 2), implying that soil enzyme activity could be used to characterize soil quality. This observation was similar to previous findings^54^. Overall, the analysis of SWC, BD, pH, soil nutrient content, and enzyme activities indicated that the basic soil conditions of artificial soils on the north-facing slopes were better than those on the south-facing slopes.

### Topographic aspect affects artificial soil structural characteristics

Soil structure is a crucial factor affecting soil function; plant, animal life, environmental quality maintenance, and soil quality^31^. Soil PSD is strongly linked to soil structure and function^55,56^. Differences were found in the PSD (clay, silt, and sand content) between two contrasting topographic aspects (Table 1). However, it is difficult to accurately determine how the topographic aspect affected the PSD as all artificial soils were sandy loam (Table 1). A previous study reported that *D*_m_ is a sensitive index that reflects the variation of soil structure^57^. Therefore, we introduced *D*_m_ to explore the effects of topographic aspect on soil PSD. In the present study, the north-facing slopes showed higher *D*_m_ values compared with the south-facing slopes, indicating that the north-facing slopes had finer soil particles. The status of soil aggregates and water-stable aggregates largely reflects soil structure and soil quality^58^. In addition, the soil structural failure rate and ∆S are two important aggregate indices that reflect the soil aggregate stability and erosion durability^2^. In the present study, the north-facing slopes had more soil aggregates and water-stable aggregates, lower soil structural failure rate and ∆S than the south-facing slopes, indicating good aggregation degree and higher aggregate stability. Overall, the topographic aspect affects artificial soil PSD, aggregate and water-stable aggregate fraction, and soil aggregate stability. A finer PSD and higher aggregate stability of artificial soils were found on the north-facing slopes. However, all of the cut slopes showed poorer aggregate degrees and aggregate stability than natural slopes.

Canopy cover can protect soil surfaces from intercepting raindrops, thereby reducing their kinetic energy and soil detachment capacity and retarding runoff formation^59^. In our study, the lower canopy cover on south-facing slopes made it more susceptible to erosion during rains, leading to the movement of fine particles and breakdown of aggregates. This observation might be the main factor that caused the differences in PSD, aggregate and water aggregate fraction, and aggregate stability between the south- and north-facing slopes. As such, large-canopy plant seeds to be sprayed onto rock-cut slope surfaces should be properly chosen to increase vegetation canopy cover. In this manner, artificial soil PSD and aggregate stability might be improved. Vegetation canopy covers should be improved for south-facing slopes. The artificial soil used in ESSS was largely composed of dispersed rock particles and had not undergone sufficient long-term soil formation processes (eluviation, deposition, transport, and biological cycles). Therefore, the viscidity between the sprayed artificial soil and the rock surface was low, thereby increasing the possibility of fine particles to be eroded after rainfall, especially on the south-facing slopes with a low vegetation canopy cover. The aggregate stability of the cut slopes was lower than that of the natural slopes because of the lack of aggregates and the viscidity between artificial soil and slope surface. We suggest improving artificial soil by enhancing the adhesion of rock particles used to compound the artificial soil or using soil that has undergone sufficient long-term soil-forming processes instead of rock particles.

### Improvements of ESSS technique

Silt, clay, and sand contents were significantly associated with SOC (Table 3), and this observation was consistent with the findings of other studies^2,31,60^. This association could be attributed to the increase in the fine fraction (clay + silt) content with a decrease in soil pore size and an increase in soil surface area. These physical and chemical properties may function as a protective mechanism against SOC from decomposing because of the isolation within and between soil micro-aggregates. Conversely, as the content of sand increases, the protective effect decreases, and the decomposition rate of SOC is accelerated^31^. Additionally, the primary mineral particles (i.e., silt, clay, and sand) and SOC were closely correlated with *D*_m_ and structural failure rate (Table 3), suggesting that these variables are crucial to controlling soil aggregation. This result was also consistent with previous findings of Chen *et al*.^5^ and Regelink *et al*.^61^. Principal component analysis concluded that these primary mineral particles and SOC were sensitive in response to soil improvement and implicated in soil formation (Fig. 7). Thus, the low percentages of clay and SOC and the interactions between them yielded two results: the aggregate stability of the south-facing slopes was less than that of the north-facing slopes, and (2) the aggregate stability of the cut slopes was less than that of the natural slopes. Therefore, two soil improvement treatments should be implemented to improve these cut slopes, especially for south-facing slopes: improve soil texture and SOC. The application of new fertilizers, such as biochar and slow-release fertilizers, can effectively amend soil texture, pore-space properties, and aggregate stability; improve nutrient use efficiency; and provide a stable and long-term supply of carbon, nitrogen, phosphorus, and other nutrients^62–66^. Therefore, adding these fertilizers onto rock-cut slopes may promote the ecological restoration of these slopes. However, the information available about these new fertilizers for rock-cut slope restoration is limited, and inorganic and soluble chemical fertilizers are sprayed onto slope surfaces for revegetation at many places^14,67–69^. Further research is needed regarding the practical application performance of new fertilizers on the ecological restoration of rock-cut slopes.

## Conclusion

This study provided a basis for exploring the effect of topographic aspect on the vegetation restoration and artificial soil quality of rock-cut slopes restored by ESSS. Our comprehensive evaluation of vegetation canopy cover, richness and diversity indices, basic soil properties and enzyme activities, fractal dimension and aggregate stability revealed that topographic aspect elicited a significant impact on the vegetation restoration and artificial soil quality of rock-cut slopes. The vegetation and soil conditions of north-facing slopes were better than those of south-facing slopes after many years of ecological restoration. The cut slopes significantly differed from natural slopes in terms of soil water content, soil organic carbon, and structural failure rate, indicating that the former has greater soil erosion potential and poorer soil condition than the latter. Overall, the application performance of ESSS on north-facing slopes was better than that on south-facing slopes, but its performance can still be improved.

Therefore, we should take the following effective measures to optimize ESSS technique: (1) considering the topographic aspect and spraying more seeds of large-canopy plants onto south-facing slope surfaces to increase vegetation canopy cover, thereby minimizing soil erosion and reducing water evaporation; (2) enhancing the adhesion of rock particles used to compound artificial soil or using soil that has undergone sufficient long-term soil forming processes instead of rock particles; (3) modifying the proportions of artificial soil components and applying new fertilizers to improve soil water content and nutrient use efficiency, thereby promoting the ecological restoration of rock-cut slopes, especially for south-facing slopes. Further studies should be performed regarding the practical application of plant species and improvement methods.
